# Motor Physical Therapy Affects Muscle Collagen Type I and Decreases Gait Speed in Dystrophin-Deficient Dogs

**DOI:** 10.1371/journal.pone.0093500

**Published:** 2014-04-08

**Authors:** Thaís P. Gaiad, Karla P. C. Araujo, Júlio C. Serrão, Maria A. Miglino, Carlos Eduardo Ambrósio

**Affiliations:** 1 Department of Physical Therapy, Faculty of Biological Science and Health, University of Jequitinhonha and Mucuri Valley/UFVJM, Diamantina/MG, Brazil; 2 Department of Surgery, School of Veterinary Medicine and Animal Science, University of Sao Paulo/USP, São Paulo, SP, Brazil; 3 Department of Biodynamic, School of Physical Education and Sports, University of São Paulo/USP, São Paulo, Brazil; 4 Department of Veterinary Medicine, School of Animal Sciences and Food Engineering, University of São Paulo/USP, Pirassununga, SP, Brazil; University of Pittsburgh, United States of America

## Abstract

Golden Retriever Muscular Dystrophy (GRMD) is a dystrophin-deficient canine model genetically homologous to Duchenne Muscular Dystrophy (DMD) in humans. Muscular fibrosis secondary to cycles of degeneration/regeneration of dystrophic muscle tissue and muscular weakness leads to biomechanical adaptation that impairs the quality of gait. Physical therapy (PT) is one of the supportive therapies available for DMD, however, motor PT approaches have controversial recommendations and there is no consensus regarding the type and intensity of physical therapy. In this study we investigated the effect of physical therapy on gait biomechanics and muscular collagen deposition types I and III in dystrophin-deficient dogs. Two dystrophic dogs (treated dogs-TD) underwent a PT protocol of active walking exercise, 3×/week, 40 minutes/day, 12 weeks. Two dystrophic control dogs (CD) maintained their routine of activities of daily living. At t0 (pre) and t1 (post-physical therapy), collagen type I and III were assessed by immunohistochemistry and gait biomechanics were analyzed. Angular displacement of shoulder, elbow, carpal, hip, stifle and tarsal joint and vertical (Fy), mediolateral (Fz) and craniocaudal (Fx) ground reaction forces (GRF) were assessed. Wilcoxon test was used to verify the difference of biomechanical variables between t0 and t1, considering p<.05. Type I collagen of endomysium suffered the influence of PT, as well as gait speed that had decreased from t0 to t1 (p<.000). The PT protocol employed accelerates morphological alterations on dystrophic muscle and promotes a slower velocity of gait. Control dogs which maintained their routine of activities of daily living seem to have found a better balance between movement and preservation of motor function.

## Introduction

Animal models for Duchenne Muscular Dystrophy (DMD) studies are used to attest the viability of therapies that aims to reduce the progression of this disease in humans. GRMD (Golden Retriever Muscular Dystrophy) is a dystrophin-deficient canine model that has been widely studied [1] since it presents muscle abnormalities that are closest to the ones seen in humans: increased creatine kinase activity, muscle hypotrophy, contractures, degeneration, endomysial and perimysial fibrosis [2], [3]. This model also presents repeated cycles of muscular necrosis and regeneration, muscle wasting, postural abnormalities and respiratory or heart failure, as seen in DMD patients [4].

Since the coding sequence of the dystrophin gene was discovered in 1987 [5], no treatment has been found to stop DMD progression. To improve quality of life and prevent complications, patients have access to supportive therapies such as physical therapy. Although these therapies cannot cure DMD, they should be well investigated as they intend to lead these patients to a better quality of life and to decrease the complications of DMD [6].

Physical therapy (PT) has been used to reduce muscular, cardiac and vascular abnormalities which develop in association with muscle strength loss [7]. The main objective of PT is the prevention of hypotrophy associated with contractures and bone deformities [8]. However, PT approaches have yielded controversial recommendations [9] and there is no consensus regarding the type and intensity of PT [10].

Muscular fibrosis is a morphological feature considered secondary to cycles of degeneration and regeneration of dystrophic muscle tissue [4]. Endomysial fibrosis is characteristic of the GRMD model, human patients and diaphragm muscle of *mdx* mouse [11], and its analysis on dystrophic muscle can bring important information about tissue adaptation. Collagen is the main component of muscle fibrosis and the types I, III and IV are the most studied types on muscular dystrophies [12]. In a previous study, we have found great amount of collagen types I and III in the endomysium and perymisium of GRMD muscle [7]. The study of localization and ratio of these types of collagen during a PT intervention can elucidate its effects on dystrophic muscle adaptation to movement.

Muscular weakness due progressive lesions in dystrophin-deficient muscular fibers leads to biomechanical adaptation and limited range of motion that impair the quality of gait. As well as DMD humans, GRMD dogs develop alterations in gait pattern. On GRMD model alterations can be visually observed from the fourth months of life of these animals [2]. Some studies on DMD patients have been conducted in early stage patients when clinical and functional evaluation do not allow to quantify initial walking worsening or to identify the changes adopted to compensate for muscle weakness [13], [14]. According to these authors, instrumented gait analysis are more sensitive than other clinical or functional assessments to verify early modifications on gait pattern of DMD humans [13] and opens new perspectives for the objective assessment of efficacy of the new therapies associated with Duchenne muscular dystrophy [14].

Previous studies with GRMD dogs have shown, mainly, the morphological and molecular features of dystrophic muscle. Assessment of muscle function was performed by functional scales [15], [16], [17] and they are important to detail phenotypic variability and progression of the disease in GRMD dogs, but they do not have the sensibility to attest small changes during the progression of the disease. Kinematic analysis of pelvic limbs joints have already been studied in this model by Barthélémy et al. [18] using accelerometer in adult animals, Marsh et al. [19] that have quantified the two-dimensional kinematics of gait during overground walking at a self-selected speed at the stifle (knee) and hock (ankle) joints. Recently, Barthélémy et al. [20] have described longitudinal ambulatory measurements of dystrophin-deficient dogs during growth and disease progression using an ambulatory gait analyzer (3D-accelerometers). In 2013, Shin et al. [21] have quantify dog gait (stride length and speed), joint angle and limb mobility (for both forelimb and hind limb), and spontaneous activity at night using a video recording system. The group of Barthélémy et al. [22] have used the accelerometer tool to assess the effects of an immunosuppressive therapy on GRMD dogs.

Until now, we have not seen the dynamics of gait using a force plate describing the gait of dystrophin-deficient dogs. We aimed here to investigate the effect of an active walking exercise protocol in dystrophin-deficient dogs using morphological analyses and kinematic and dynamic quantitative assessment tools.

## Methods and Ethics Statement

Four dystrophic dogs were selected from GRMD kennel – Brazil. Because management of a GRMD model colony is expensive, laborious, and this disease is rare, we have chosen to study a reduced number of animals which had in common the same age. The use of dystrophic animals of the same age decreases phenotypic variability that is a usual feature of this model [2], [15]. Research was approved by the Bioethics Committee of the School of Veterinary and Animal Science of the University of São Paulo, protocol n° 1030/2006. DNA analysis on blood samples from these dogs were done to confirm the presence of muscular dystrophy. Prior to data collection, all dogs had a complete clinical, orthopedic and neurologic assessment to ensure that there were no underlying conditions that would influence gait.

### Study design

Assessments were performed at time zero (t0) and time one (t1). At t0, animals were 5 months old (mean height: 43 cm ±1.15; body mass: 12.6 kg ±1.9). During 12 weeks, two GRMD animals underwent exercise training and were named TD (Treated dog 1 – TD1 and Treated dog 2 – TD2). Other 2 dogs had maintained their routine of activities of daily living and were named CD (Control dog 1 - CD1 and Control dog 2 - CD2). An open-air enclosure of 20 m^2^ was available for all GRMD dogs during the day, while at night, the same dogs were kept in closed pens of 1 to 3 m^2^. Time spent in these places during day and night was the same for all GRMD dogs, excepted when TD were undergoing exercise training. Daily activities consisted of feeding, cleaning care and clinical analysis. Evaluations at t1 were performed after these 12 weeks when animals were 9 months old (mean height: 51.1±0.85; body mass: 17.8±2.24).

### Intervention

Type, frequency and duration of the physical therapy protocol were selected from published recommendations for DMD patients [23], [24], [25], [26] and adapted to GRMD dogs. These publications bring indicated modalities of exercise based on previously investigation on animal models for DMD and on human studies (See Table S1). Motor physical therapy (PT) sessions were performed 3 times a week, with a resting day between PT sessions, for 40 minutes/day over 12 weeks. An area with 36×5 meters was constructed for PT session. PT sessions consisted of walking exercise with velocity defined as at least 10% more than their walk velocity. During PT sessions, velocity was recorded using a chronometer and dogs were allowed to rest between each circuit, aiming to avoid fatigue. The velocity was archived using the chronometer and calculated based on distance covered. Velocity had been recorded during the walking period and stopped during the rest time. TD left the Zero Point and walked 8 times the 36 meters area. Each circuit (36 meters) was considered valid if animals had completed it and its minimal exercise velocity established. Morphological and biomechanics of gait data from GRMD dogs were collected at t0 and t1.

### Immunohistochemistry (IHC)

Muscle fragments were collected from *biceps femoralis* of each dog for morphological analysis. To avoid any loss of motor function, a single muscle was chosen to represent the pelvic limb. Hind limbs are considered more affected in the canine dystrophy model [27] and *biceps femoralis* was choosen to represent pelvic limb once it is a large and superficial muscle that provides easy access during muscle collection. An open biopsy was performed with the collection of a 1 cm^2^ fragment from the middle portion of the muscle. Dogs were submitted to an anesthetic protocol previously approved by the Ethics Committee. In order to avoid scar fibrosis after the first biopsy, the second one was performed at least 2 centimeters away from the scar tissue. Muscle fragments were fixed in Paraformaldehyde solution at 4%, treated with increasing ethanol series (70 to 100%) to dehydration and xylene for clearing. Fragments were embedded in paraffin and sections of 5 µm were obtained.

Primary antibodies against collagen (anti-mouse) types I and III (Calbiochem) (1∶500 and 1∶750 dilution) were applied on muscle sections, separately. After being washed three times in phosphate-buffered saline (PBS), the sections were incubated for 45 minutes with biotinylated antibody (Dako Ltd.) in a damp chamber at room temperature. After three more rinses in PBS, streptavidin was applied for 45 minutes. The sections were washed with PBS and covered with 3,3′-diaminobenzidine tetrahydrochloride (DAB) for 1 to 2 minutes. The primary antibody was omitted in control sections. Photomicrographs were taken using an optical microscope (Axioscope, Zeiss) equipped with an Olympus BX 60 camera.

### Gait analysis

Data was collected at the Biomechanics Laboratory of the School of Physical Education and Sports of the University of São Paulo. Video records of the gait and Ground Reaction Forces (GRF) collection were synchronized collected. Aiming that animals presented a naturally way of walk during data collection, GRMD dogs had gone to the Biomechanics Laboratory for 3 times, for one hour, during one week to adaptation to the environment prior to t0 collection.

### Video collection

Digital video (Panasonic PV-GS50S model) of sagittal plane of motion was collected during over-ground walking at the dog's self-selected pace with a single camera. The camera was positioned at the transversal plane at the cranio-caudal direction at the right side four meters away of the animals. Peak Motus System (Peak Performance Technologies, Inc) has been used to register the position and displacement of each body segment during gait.

**Figure 1:**
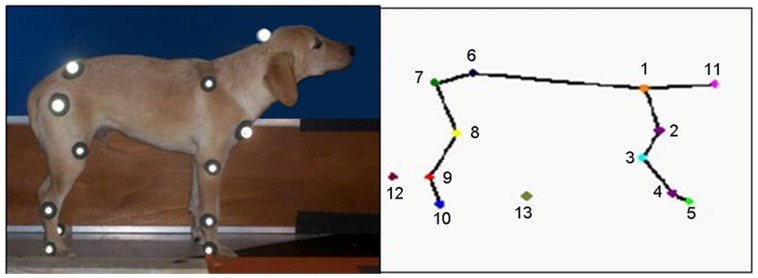
Location of anatomical landmarks. 1.*spina scapulae*/acromion; 2.humerus *tuberculim majus*; 3.humerus *epicondylus lateralis*; 4.ulna *processus estyloideus*; 5. caput of *ossa metacarpalia* V; 6.crista iliaca; 7. femur *trochanter major*; 8.femur *condylus*; 9. *malleulus lateralis* and 10. caput *ossa metatarsalia* V. 11. occipital crest, 12. distomedial aspect of the second contra-lateral metatarsus and 13. disto-medial aspect of the second contra-lateral metacarpus.

Thirteen retro-reflective markers (3M) were placed on anatomical landmarks of the right limbs of the animals: 1.*spina scapulae*/acromion; 2.humerus *tuberculim majus*; 3.humerus *epicondylus lateralis*; 4.ulna *processus estyloideus*; 5. Caput of *ossa metacarpalia* V; 6.crista iliaca; 7. Fêmur *trochanter major*; 8.femur *condylus*; 9. *Malleulus lateralis* and 10. Caput *ossa metatarsalia* V. Three other landmarks were placed at occipital crest, disto-medial aspect of the second contra-lateral metatarsus and disto-medial aspect of the second contra-lateral metacarpus (Figure 1). To facilitate marker placement, trichotomy were made around each anatomical landmark and glue was used to fix the markers of 1 to 2 cm^2^. Dogs were placed in lateral recumbency to landmarkers attachment. Following marker application, manual flexion and extension of the joints after application of the markers was used to verify that the marker positions were as close as possible to the joint centers.

### Video processing

Each one of the trials were identified by three investigators as valid or not. Trials were considered valid if dog walked over the force plate without deviation from the plane. Only data from the right side of the animals were considered for analysis. Six valid trials of each one of the dogs at t0 and t1 were identified for further analysis. A complete gait cycle was defined as starting the contact with the fingers of the right forelimb and ending with the ipsilateral hindlimb contact. A rest period after each one of the cycles was provided, if necessary, for all GRMD dogs. Dogs were allowed to rest for a maximal time of 3 minutes between each cycle. Digitizing software Peak Motus System and MATLAB (The MathWorks, Natick, MA) programs were used to calculate joint angles of the shoulder, elbow, carpus, hip, stifle and tarsus. Raw x–y co-ordinate data were filtered using a Butterworth filter with a cutoff frequency of 4 Hz. Each gait cycle was normalized in time (100% of gait cycle) and body mass (N/kg).

### Dynamics of gait

A quartz crystal piezoelectric Kistler force plate (FP) (Kistler type 9287, Kistler Instrumente AG, Winterthur, Switzerland) was used together with the Kistler 9865B charge amplifiers. The FP (60×90 cm) was mounted flush with the surface. The middle 2 m of the runway was bordered behind by a 50-cm-high fence at the left side of the dogs to guide the dogs over the FP. The sampling rate was 100 Hz, and recordings were saved for further processing. Mediolateral (Fz), cranio-caudal (Fx), and vertical (Fy) forces were analyzed. Fy was calibrated with a standard weight before each recording session.

### Statistical analysis

Statistical analysis was performed using software Biostat (Biostat 2009 version 5.3.5 for Windows). Kolmorov-Smirnov test was used to determine if mean data of angular displacement, GRF and velocity were normally distributed. Data was not normally distributed and Wilcoxon test was applied to attest individually difference between t0 and t1 of each one of the variables. Each dog had its all data at t0 compared to all data at t1 for each one of the studied variables.. All data are expressed as mean±SD. The level of significance was set at p<.05.

## Results

**Figure 2:**
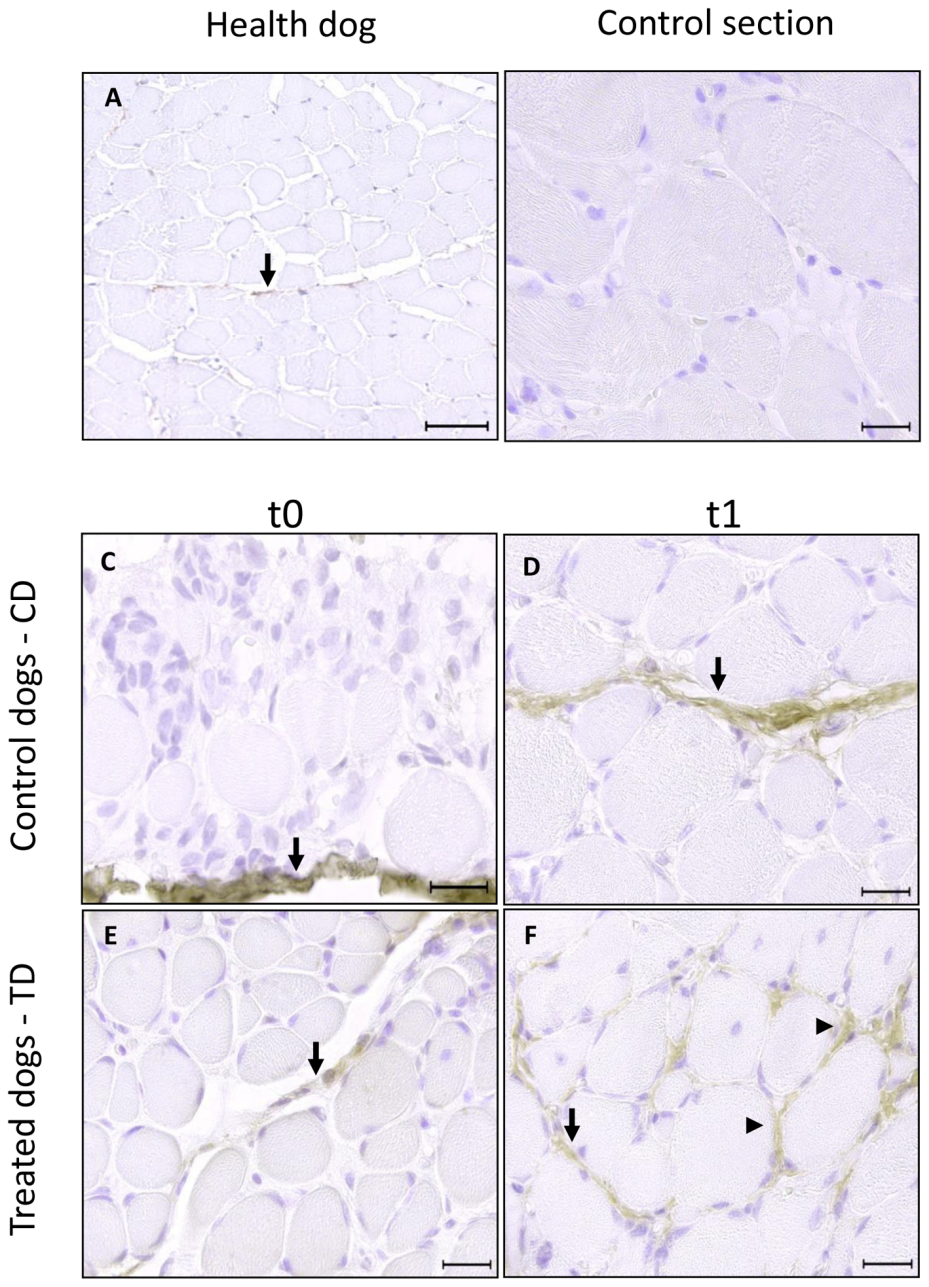
Photomicrographs of immunohistochemical analysis of collagen I in skeletal muscle. Positive immunostaining for collagen type I (1∶500) in *biceps femoralis* of healthy dog (A), bar = 50 µm; control section, bar = 20 µm; CD at t0 (C) and t1 (D), TD at t0 (E) and t1 (F), bar = 20 µm. 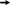 perimysial fibrosis and ▸ endomysial fibrosis.

**Figure 3:**
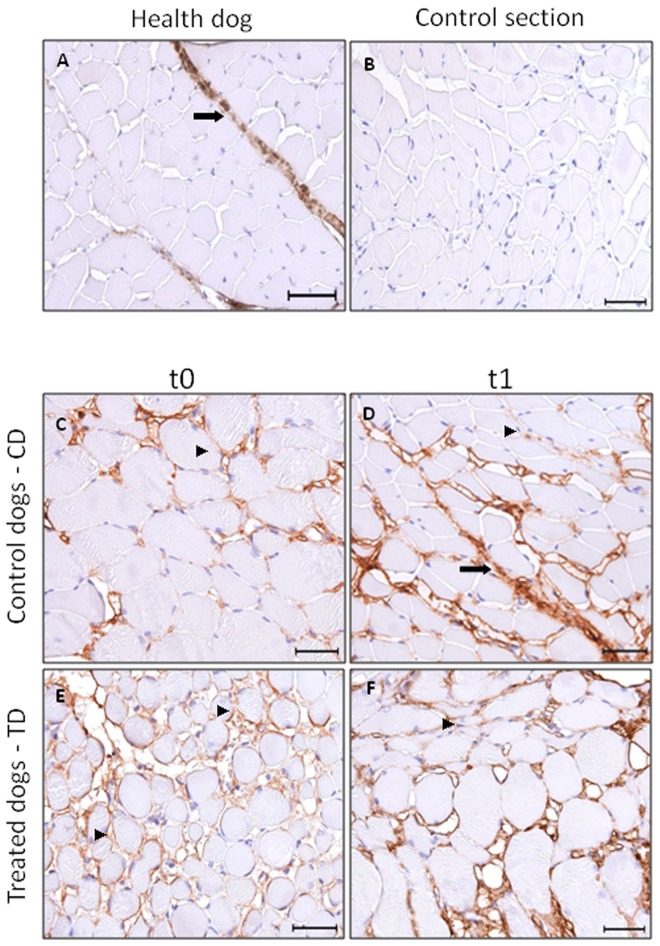
Photomicrographs of immunohistochemical analysis of collagen III in skeletal muscle. Positive immunostaining for collagen type III (1∶750) in *biceps femoralis* of healthy dog (A), bar = 50 µm; control section, bar = 40 µm; CD at t0 (C) and t1 (D), TD at t0 (E) and t1 (F), bar = 40 µm. 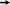 perimysial fibrosis and ▸ endomysial fibrosis.

Immunohistochemistry analysis at t0 has shown that collagen I is present in greater amount at perymisium of TD and CD than healthy dogs. At t1, type I collagen was also observed at endomysium of both TD muscle (Figure 2) and no positive signal at CD. Collagen type III was observed at perymisium and endomysium of TD and CD from t0, and did not change between t0 and t1 (Figure 3).

**Figure 4:**
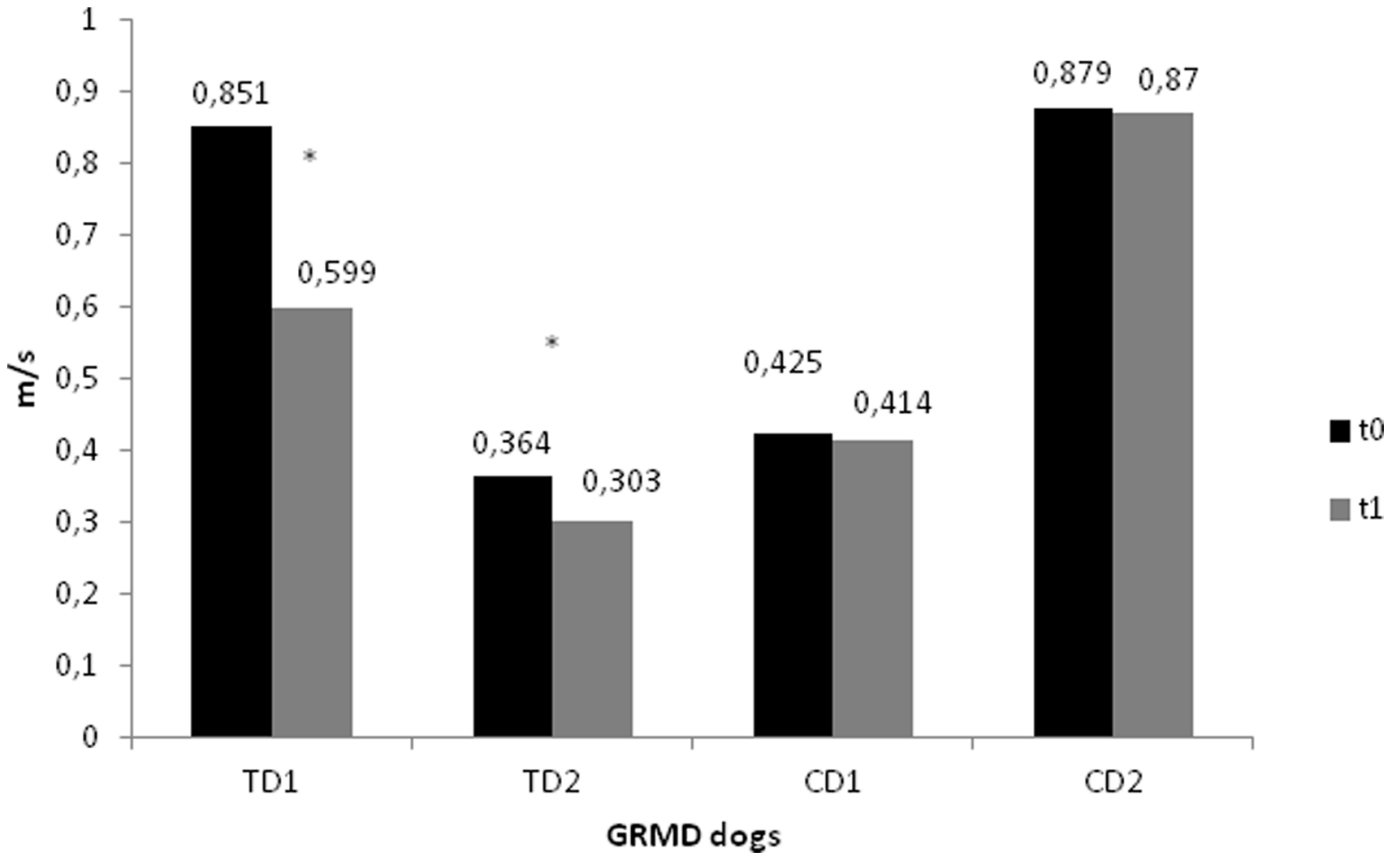
Data of gait speed of biomechanical analysis of TD1, TD2, CD1 and CD2 at t0 and t1. Values of TD1 and TD2 had presented difference between t0 and t1, p<.05. * denote significant differences between t0 and t1, with p<.05.

**Figure 5:**
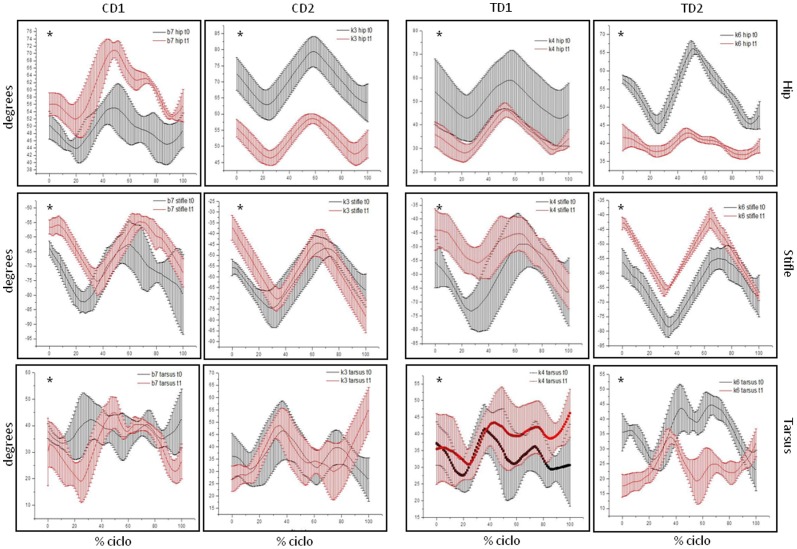
Mean and standard deviation of angular displacement (degrees) of hindlimbs. Hip, stifle and tarsus of TD1, TD2, CD1 and CD 2at t0 and t1. Images with * denote significant differences between t0 and t1, with p<.05.

**Figure 6:**
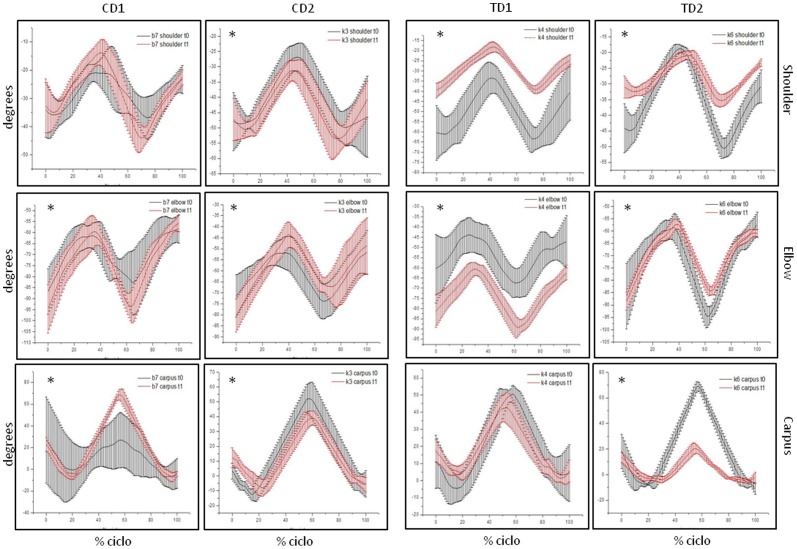
Mean and standard deviation of angular displacement (degrees) of forelimbs. Shoulder, elbow and carpus of TD1, TD2, CD1 and CD2 at t0 and t1. Images with * denote significant differences between t0 and t1, with p<.05.

**Figure 7:**
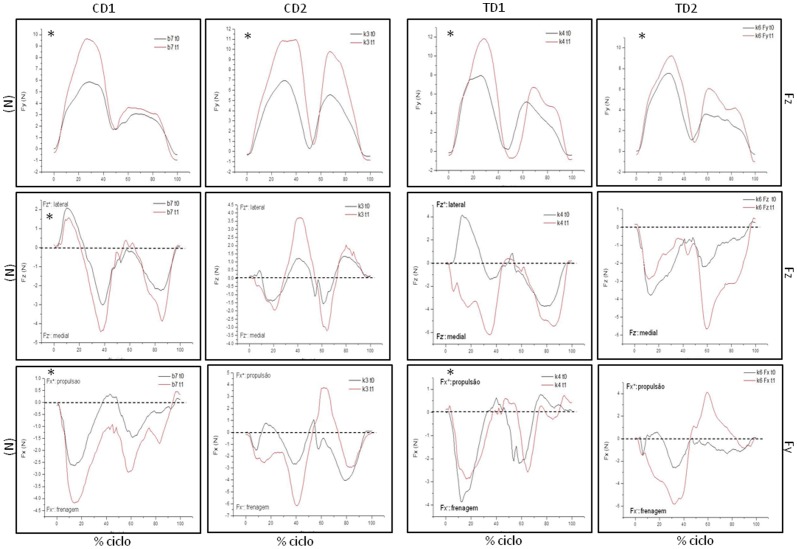
Mean and standard deviation of GRF: vertical (Fy), craniocaudal (Fx) and mediolateral (Fz) of TD1, TD2, CD1 and CD2 at t0 and t1. Data in Newton (N) was normalized by body weight. The first 50% of the gait cycle represents forelimb contact and the last 50% represents the hindlimb contact with the FP. Images with a * denote significant differences between t0 and t1, with p<.05.

Velocity of gait of TD1 and TD2 have been slower at t1 than at t0 (p<0.000). Data of mean velocity of gait of each one of the dogs at t0 and t1 are shown at Figure 4 and detailed on Table 1. CD1 and CD2 have maintained their gait speed between t0 and t1 (Figure 4). Results of angular displacement (Figures 5 and 6) and GRF (Figure 7) were described individually for each joint and dogs at t0 and t1. Data are presented as average of six trials of each dog. Data of CD at t1 have been shown to illustrate the progression of dystrophin deficiency without exercise intervention.

**Table 1:** Standard velocity of gait of each one of the dogs at t0 and t1.

		TD1	TD2	CD1	CD2
**t0**	mean ± sd	0.851±0.4	0.368±0.09	0.425±0.30	0.879±0.32
	min-max (m/s)	0.18–2.19	0.01–1.50	0.10–0.98	0.19–2.19
**t1**	mean ± sd	0.599±0.22	0.302±0.12	0.414±0.18	0.87±0.31
	min-max (m/s)	0.06–1.84	0.01–1.30	0.08–1.26	0.16–2.38

t0 = time zero (5 months old); t1 = time one (9 months old); sd = standart deviation; min = minimal velocity; max = maximal velocity.

### Control Dog 1 (CD1)

All joints, with the exception of the shoulder (p = 0.215), have changed from t0 to t1 (p<0.000). Hip, stifle and carpal joints have presented an extended feature at t1. All GRF have changed from t0 to t1 (p<0.000). Vertical force (Fz) of forelimb and braking force (Fx^−^) increased from t0 to t1. CD1 has directed its hindlimb force (Fy) to the medial direction at t1.

### Control Dog 2 (CD2)

All joints, with the exception of the tarsus (p = 0.482), have changed from t0 to t1 (p<0.000). Hip joint had an important flexed feature at t1 when compared to t0. Vertical force values had showed a significant increase in forelimb and hindlimb (p<0.000). Fy and Fx had maintained its values between t0 and t1.

### Treated Dog 1 (TD1)

All joints, with the exception of the carpal joint (p = 0.474), have changed from t0 to t1 (p<0.000). At t1, TD1 walked with a hip and elbow joint more flexed than at t0. On the other hand, stifle, tarsus and shoulder have presented an extended feature during walking. Fz and Fy have presented significant difference between t0 and t1 (p<0.000). Vertical force (Fz) has increased from t0 to t1 and TD1 has directed its fore and hindlimb force (Fy) to the medial direction.

### Treated Dog 2 (TD2)

All joints of TD2 have changed from t0 to t1 (p<0.005). Hip and carpal joints have presented an important decrease in their ROM. At t1, these joints and the tarsal one have presented a flexed feature during gait. Stifle joint had presented an extended feature at t0. Vertical force (Fz) has increased from t0 to t1 and TD2 has directed its hindlimb force (Fy) to the medial direction.

## Discussion

PT of active walking exercise, three times per week, during 40 minutes, with intervals of rest aiming to avoid fatigue between days and during exercise protocol, has decreased gait velocity and increased the amount of collagen type I at endomysium of dystrophic dogs. This same protocol has not influenced angular displacement or GRF of dogs. Control dogs that have maintained their daily life activities, e.g. postural changes independently with the objective of getting water and food, with more hours of rest during the week, have reached the balance between movements without aggregate muscular damage.

Fibrosis is poorly understood on dystrophic muscle, but it seems to be the consequence of inflammatory infiltrate due to muscular damage [28]. Our results have shown that skeletal muscle of GRMD animals at t0 have already presented increased muscular fibrosis deposition when compared to skeletal muscle of healthy dogs [7]. At t0, collagen type I was immunolocalizated in a large amount on perymisium of GRMD dogs and collagen type III was immunolocalizated in large amount on endomysium of GRMD dogs. This finding is in accordance with Cozzi et al. [29] and Nguyen et al. [11] who have found the presence of increased amount of fibrosis in early ages in dystrophic animals.

Collagen type I was influenced PT protocol and showed that treated dogs presented higher muscular damage. A longitudinal study with 25 human DMD patients have shown that among histopathological features, myofiber atrophy, necrosis, and fatty degeneration, only endomysial fibrosis was correlated with poor functional outcome assessed by muscle strength and age of loss of ambulation [30]. Collagen type I is characteristic of tissue with low flexibility, e.g. tendon. The presence of this type of collagen at the endomysium of dystrophic dogs who underwent walking exercise protocol suggests a functional loss of these muscles.

Some authors suggest that the presence of collagen I in muscular dystrophy inhibits muscle regeneration and support the production of more muscular collagen [31]. Both mechanisms affect strategies of treatment of DMD. Drugs or therapies able to reduce fibrosis [28], such as antifibrotic therapy [32], have been shown to be extremely promising to DMD patients by enhancing the grip strength of pelvic limbs [33] or improving cardio respiratory function [34].

Until now, we have seen few recent published papers that have used biomechanical tools to asses gait variables of GRMD model. Barthelémy et al. [18] have investigated the viability of accelerometer as a quantitative tool to assess the gait of adult dystrophic dogs. Marsh et al. [19] showed the kinematic features of pelvic limbs from GRMD and healthy Golden Retriever females. Another study of Barthélémy et al. [20] have followed ambulatory measurements of gait abnormality in dystrophin-deficient dogs from 2 to 9 months of age. Biomechanical assessment of gait, have been used by Barthélémy et al. [22] to attest effects of an immunossuppressive therapy. We have used kinematics and dynamics of gait to attest the progression of four GRMD dogs from 5 to 9 months of age with and without the presence of an active walking protocol of physical therapy.

At t0, mean velocity of all dogs (0.63 m/s ±0.27) were lower than healthy dogs and considered a slow velocity of gait. Gait speed is related to the time that limbs are in contact with the ground while walking. Exercise could have provoked a decrease in gait speed which means a deterioration of the gait function. Mean velocity of dystrophic dogs in the study of Barthélémy et al. [18] was 0.72 m/s, similar to the one observed in our dogs and significantly lower than their control healthy dogs (3.97 m/s). The gait of TD was more difficult to be performed than the gait of CD at t1. It is possible that fatigue may be the reason behind this reduced velocity, but studies to investigate muscular activation using electromyography would be necessary to conclude about this theory. PT played an important role in the decreasing velocity of walking and it also clinically reflects in functional deterioration of the muscle.

Elbow, hip and stifle joint showed significantly changes from t0 to t1 in all GRMD dogs, but PT has not influenced this data. These changes reflect the natural progression of muscular dystrophy. The stifle joint has shown an extended feature at t1 for all dogs, which are in accordance with visual assessment previously described using a physical exam score [15]. The stifle joint also presents less flexion movement at t1 compared to t0, when maintaining an extended position. Marsh et al [19] have studied six GRMD dogs and have found that they have presented the stifle joint relatively more extended during walking. At the hock joint, GRMD dogs displayed less range of motion and walked with the joint relatively less flexed compared to controls.

Elbow and hip joints are also related to disease progression and until now have been poorly studied [1], [2] most likely due the difficultly of visual observation of the proximal joint during gait. According to Van der Walt [35], the hip is an important joint to understand muscle skeletal alteration on quadrupeds because 30% of orthopedic diseases affect pelvic limbs. Elbow, hip and stifle joints should be better investigated on GRMD model.

Shoulder, carpal and tarsal joints have also changed from t0 to t1 but these results were not common to all dystrophic dogs and have not been influenced by PT. Figures 5 and 6 show that they change in an individual manner. Once an animal has a quadruped gait, changes in one joint alter the weight distribution on whole body and cause adaptation in the other joints. It is possible that each dog developed adaptations that are related to the extent and distribution of their first signs of muscle skeletal alterations.

Vertical force (Fy) has increased from t0 to t1 in treated and control dogs. Fy were already higher at t0 when compared to healthy dogs [36]. It suggests that dystrophic dogs present an overload to maintain their gait, most likely because of loss of muscle mass and strength. Healthy dogs present increased Fy on thoracic limbs when compared to pelvic limbs [36], [37], [38]. This feature was also observed in our dystrophic dogs during the entire study, which indicates that even with adaptations, GRMD still maintain this distribution of body weight between thoracic and pelvic limbs.

Data of cranio-caudal force (Fx) indicate that propulsion force is practically absent on these animals. Among quadrupeds, thoracic limbs are responsible for braking force and pelvic ones are responsible for the propulsion force during gait [36], [39]. Decreased values suggest loss of strength in these limbs [40]. On muscular dystrophy, progressive loss of strength which initiates at pelvic limbs leads propulsion force practically absent already at five months of age. This feature was maintained along this study and has not suffered influence of PT.

As DMD is a progressive disease which presents its first symptoms early in life, physiotherapist can increase quality of life of patients offering clarification and information to caregivers and family. Moreover, it is possible to maintain muscle flexibility stimulating active movements while playing with kids or stimulating the independent realization of activities of daily life. Physiotherapists have great concern on motor objectives, but the contradiction of movement *versus* motor function still needs clarification. Physical therapy protocol of active walking exercise, 3 times per week, during 40 minutes, with intervals of rest, has shown negatively effects on gait speed and has increased the amount of muscular collagen of a type poorly flexible (type I) of dystrophic dogs. Our results here described in association with the literature allow us to suggest that DMD patients should be encouraged to perform their daily activities independently and it should be supported by adaptations in their environment and individual limitations should be respected. Physiotherapists should also take into account the expectation of patients and their interest on therapy when planning therapies sessions. Nowadays, there is a lot of research on drugs, cellular or gene therapies in pre-clinical models of DMD [41], [42] aiming to ameliorate or, at least, decrease the velocity of progression of the disease. At the moment, professionals involved in therapies of support, such as physiotherapists, must offer the best that can be done for patients and their families. Physiotherapists also must know how to measure the movement that is possible, and find a balance between minimal muscular lesion and the preservation of motor, mental and vital function of patients.

## Supporting Information

Table S1 **All published articles cited on this table reported that limited research has been carried out on the type, frequency, and intensity of recommended exercise prescription for Duchenne Muscular Dystrophy human patients.**
(DOC)
