# Supplementary material for: Motor Physical Therapy Affects Muscle Collagen Type I and Decreases Gait Speed in Dystrophin-Deficient Dogs
Source: PLoS One. 2014 Apr 8;9(4):e93500. doi: 10.1371/journal.pone.0093500 (PMC3979701; doi:10.1371/journal.pone.0093500)
Supplement: Table S1 — All published articles cited on this table reported that limited research has been carried out on the type, frequency, and intensity of recommended exercise prescription for Duchenne Muscular Dystrophy human patients. (DOC) [file pone.0093500.s001.doc]

|  | Published recommendations of physical therapy for DMD  (first author and number of citation) | | | |  |
| --- | --- | --- | --- | --- | --- |
|  | Eagle [23] | Ansved [24] | Bushby [25] | Cup [26] | PT intervention on GRMD dogs |
|  |  |  |  |  |  |
| Type | 1.Encourage voluntary active exercise  2.Eccentric activities should be avoided. | 1. low-resistance training are indicate  2. High-resistance and eccentric training should be avoided | 1. sub maximum (gentle) functional strengthening/activity for ambulatory patients or in the early non-ambulatory stage    2. High-resistance strength training and eccentric exercise are inappropriate | Strengthening  exercises in combination with aerobic exercises have positive effect on dystrophic muscle | Active walking exercise which had a minimal velocity of 10% over the velocity of free walking. Minimal velocity ensures that intervention was of enough intensity to provide a training stimulus. |
| Frequency |  | Beneficial effects of adopting an active lifestyle |  | Frequency of 3 to 5 days a week. | 3 times per week, during 40 minutes, allows one day of rest between each day of intervention aiming to avoid fatigue between days and during exercise protocol. |
| Duration | Active exercise on a regular basis to  promote general physical health to be encouraged |  |  | For both muscle strengthening and aerobic exercises the  entire program should last at least 10 weeks | 12 weeks of intervention |

Table S1: All published articles cited on this table reported that limited research has been carried out on the type, frequency, and intensity of recommended exercise prescription for Duchenne Muscular Dystrophy human patients.
